# Combination therapy with anti-PD-1 antibody, radiotherapy, and tyrosine kinase inhibitor for unresectable primary ectopic hepatocellular carcinoma: a case report with genomic profiling and literature review

**DOI:** 10.3389/fimmu.2026.1746990

**Published:** 2026-07-14

**Authors:** Peng Tang, Weixing Liu, Yating Xu, You Long, Yixiao Li, Jiaxin Li, Mingheng Liao, Xin Wang, Jin Zhou, Yong Zeng

**Affiliations:** 1 Department of Liver Surgery, West China Hospital, Sichuan University, Chengdu, China; 2 Department of Pancreatic Surgery, West China Hospital, Sichuan University, Chengdu, China; 3 West China School of Medicine, Sichuan University, Chengdu, China; 4 Department of Radiation Oncology, West China Hospital, Sichuan University, Chengdu, China

**Keywords:** case report, genomic profile, immunotherapy, multidisciplinary team, unresectable ectopic hepatocellular carcinoma

## Abstract

**Background:**

Ectopic hepatocellular carcinoma (EHCC) is an exceedingly rare malignancy characterized by its occurrence outside the liver without a detectable intrahepatic primary tumor. Owing to its rarity and the absence of standardized management guidelines, the diagnosis and treatment of EHCC pose significant challenges.

**Methods:**

We report a case of unresectable, multifocal EHCC managed by a multidisciplinary team (MDT). Comprehensive molecular profiling, including whole-exome sequencing (WES) and RNA sequencing (RNA-seq), was performed to characterize the tumor’s genetic and transcriptomic landscape. A systematic literature review of EHCC case reports in the PubMed database was also conducted.

**Results:**

A patient in his sixties was diagnosed with unresectable, multifocal EHCC localized within the abdominal cavity. The diagnosis was confirmed by laparoscopic biopsy and histopathological examination, which revealed hepatocellular carcinoma with fatty metaplasia, corroborated by positive immunohistochemical staining. Comprehensive genomic analysis using RNA-seq and WES identified key molecular features potentially involved in EHCC pathogenesis. Guided by the MDT, the patient received a multimodal regimen comprising lenvatinib (8 mg once daily), camrelizumab (200 mg every two weeks; an anti-PD-1 antibody), and stereotactic body radiotherapy (SBRT) targeting residual abdominal lesions. The patient achieved a progression-free survival (PFS) of 27 months and an overall survival (OS) of 30 months. A systematic review of 55 reported EHCC cases provided a comprehensive overview of the disease’s demographic, clinical, management, and prognostic characteristics.

**Conclusion:**

This study provides the molecular profiling of an EHCC case, offering descriptive insights into its genomic and transcriptomic landscape. Furthermore, the favorable clinical outcome associated with the combination therapy of lenvatinib, camrelizumab, and SBRT suggests the potential value of this multimodal strategy, indicating a potential therapeutic approach for future cases.

## Introduction

Hepatocellular carcinoma (HCC) is the most common primary liver malignancy and the fourth leading cause of cancer-related mortality worldwide (1–4). Although the majority of HCC cases originate within the liver, exceedingly rare cases have been reported of HCC arising in extrahepatic sites—including the abdominal cavity, peritoneum, or chest wall—in the absence of a detectable primary liver lesion (5, 6). This rare entity is termed ectopic hepatocellular carcinoma (EHCC).

The rarity of EHCC poses significant diagnostic and therapeutic challenges, as no internationally recognized management guidelines currently exist. Most reported cases of EHCC are solitary tumors that are amenable to radical resection and are associated with a favorable prognosis (6, 7). However, the prognosis remains poor for patients with unresectable EHCC, characterized by multiple disseminated or locally advanced lesions (8–10). Given the complexity of therapeutic decision-making in advanced HCC, management by a multidisciplinary team (MDT) is recommended to ensure individualized, stage-appropriate care (11–13). MDT-based care has been associated with improved survival in patients with intermediate-to-advanced HCC (14). Recently, the therapeutic landscape for advanced HCC has expanded to include combination strategies integrating tyrosine kinase inhibitors (TKIs), immune checkpoint inhibitors (ICIs), and locoregional therapies such as stereotactic body radiotherapy (SBRT) or transarterial chemoembolization (TACE). The efficacy and safety of these combination therapies for advanced HCC have been demonstrated in multiple clinical trials (15–18). However, the application of these strategies in the management of EHCC has not been extensively explored and warrants further investigation.

We present a rare case of unresectable, multifocal EHCC originating in the abdominal cavity. Comprehensive genomic profiling was performed using RNA sequencing (RNA-seq) and whole-exome sequencing (WES). The analysis revealed significant upregulation of the interferon response pathway, a factor that may be critical for EHCC pathogenesis and could inform novel therapeutic strategies for this rare condition. To our knowledge, this is the first reported case in which combination therapy with lenvatinib, camrelizumab (an anti-PD-1 antibody), and SBRT was employed, resulting in a progression-free survival (PFS) of 27 months and an overall survival (OS) of 30 months. This case report was prepared in accordance with the CARE guidelines (19).

This case provides valuable insights into the genomic profile of EHCC and highlights the potential efficacy of such combination therapies for this rare disease.

## Case presentation

A patient in his sixties was incidentally found to have abdominal lesions during a routine examination at a local clinic, which was performed due to facial palsy. He was subsequently referred to our hospital for further evaluation. The patient reported no abdominal pain, discomfort, fatigue, diarrhea, constipation, vomiting, nausea, jaundice, or weight loss. Physical examination revealed a soft, non-distended abdomen without tenderness or rebound. No palpable masses were detected, and neither the liver nor spleen was palpable below the costal margin. The patient had no history of hepatitis, cirrhosis, diabetes, hypertension, cardiovascular disease, or cerebrovascular disease, and denied any family history of tumors or hereditary disorders. No specific interventions had been administered prior to admission.

Laboratory tests revealed normal blood cell counts and liver function (Child-Pugh class A, score: 5). However, serum levels of alpha-fetoprotein (AFP) and protein induced by vitamin K absence or antagonist-II (PIVKA-II) were mildly elevated, at 13.20 ng/mL (normal ≤7 ng/mL) and 350.00 mAU/mL (normal 6–32.5 mAU/mL), respectively. Contrast-enhanced magnetic resonance imaging (MRI) revealed heterogeneous signal intensity shadows in the superior segment of the right liver lobe and beneath the diaphragm, with similar abnormalities surrounding the spleen. The largest cross-sectional lesion measured approximately 12.2 × 1.5 cm. The lesions were composed predominantly of adipose tissue, while the solid components demonstrated enhancement on the contrast-enhanced phases (**Figure 1A**). To further characterize the findings, the patient underwent contrast-enhanced computed tomography (CT), which also revealed patchy, cotton-wool-like mixed-density shadows in the right subdiaphragmatic region and the right perihepatic space. These lesions were primarily composed of adipose tissue interspersed with solid components, with similar lesions noted around the spleen (**Figure 1B**). However, comprehensive evaluation identified no abnormal lesions within the liver parenchyma itself. Based on the above examinations, the initial diagnosis was lipoma or liposarcoma, potentially originating from the retroperitoneum or peritoneum.

**Figure 1:**
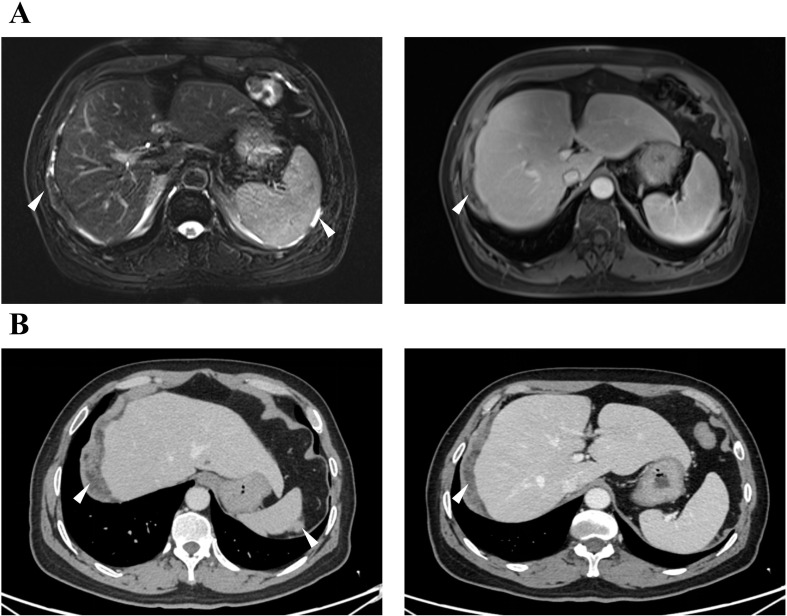
Pre-treatment abdominal imaging findings. **(A)** Contrast-enhanced MRI demonstrates mixed signal intensity lesions in the right hepatic subdiaphragmatic region and perisplenic area. **(B)** Contrast-enhanced CT reveals patchy, cotton-like mixed-density shadows in the right subdiaphragmatic space, right perihepatic region, and around the spleen. △, Tumor

Preoperatively, following a comprehensive multidisciplinary discussion, diagnostic laparoscopy was scheduled. The surgical strategy prioritized the patient’s explicit preference for the preservation of normal hepatic parenchyma. Intraoperative exploration revealed multiple xanthomatous, cloud-like neoplasms infiltrating the diaphragm, round ligament of the liver, abdominal walls, and pelvic cavity. Given the diffuse infiltrative growth pattern which precluded an R0 resection, a planned laparoscopic biopsy was performed. The postoperative course was uneventful, and the patient was discharged on the second postoperative day.

Pathological examination revealed three key diagnostic features: (1) lipid-laden macrophages, (2) proliferative adipocytes with focal lipoblastic differentiation, and (3) dense histiocytic infiltrates (**Figures 2A, B**). Immunohistochemical staining was positive for CK8/18, HepPar-1, GPC-3, Arg1, HSP70, and Ki-67 (labeling index 30%) (**Figures 2C–H**), and negative for PCK, EMA, S100, CDK4, MDM2, CD1α, HMB45, CR, KP-1, PGM-1, CD34, and AFP. Fluorescence *in situ* hybridization (FISH) showed no amplification of the *MDM2* or *CDK4* genes. Collectively, these findings confirmed the diagnosis of hepatocellular carcinoma with prominent steatosis (see **Supplementary Materials** for detailed immunohistochemical staining method).

**Figure 2:**
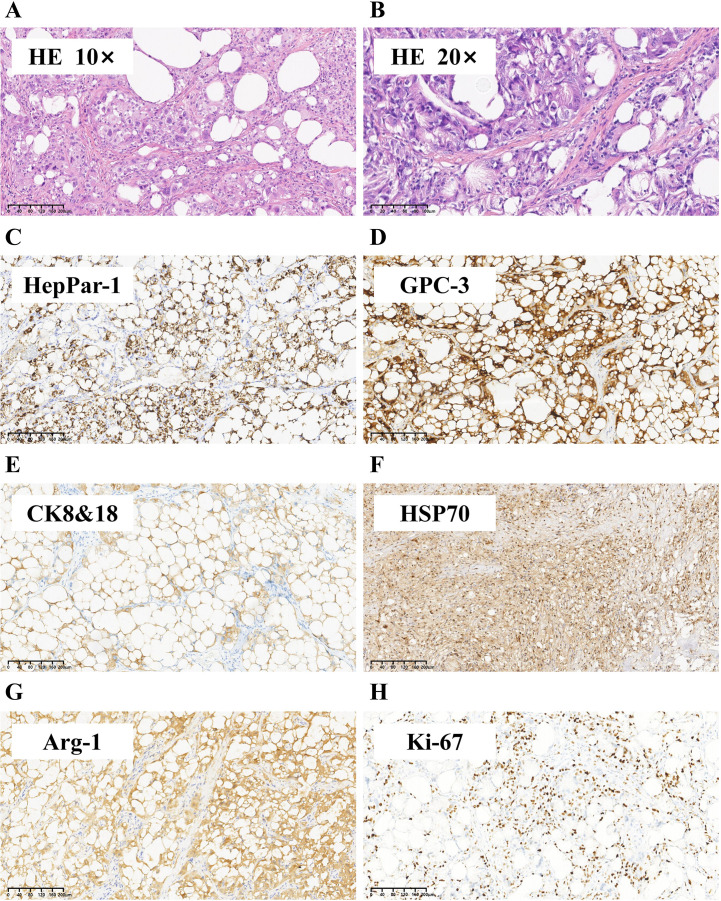
Histopathological and immunohistochemical features. **(A, B)** Hematoxylin and eosin (H&E) staining (A:10×; B: 20×) demonstrates lipid-rich mononuclear cells, proliferative adipocytes/lipoblasts, and abundant histiocytes. **(C–H)** Immunohistochemical analysis reveals positivity for **(C)** HepPar-1 (10×), **(D)** GPC-3 (10×), **(E)** CK8/18 (10×), **(F)** HSP70 (10×), **(G)** Arg-1 (10x), and **(H)** Ki-67 (30% positivity, 10×).

Following the MDT confirmed diagnosis of EHCC, the patient initiated systemic therapy one month postoperatively, comprising lenvatinib (8 mg QD) and camrelizumab (200 mg Q2W). Three months after treatment initiation, a contrast-enhanced CT scan was performed to evaluate the treatment response. Imaging revealed a significant reduction in the abdominal lesions, achieving partial response (PR) according to RECIST 1.1 criteria, with unremarkable liver parenchyma (**Figure 3A**). Treatment was therefore continued, with quarterly follow-up visits scheduled, which included a complete blood count, liver function tests, tumor marker analysis, and contrast-enhanced abdominal CT. Nearly one year after initial treatment, a slight elevation in PIVKA-II levels was observed; Although imaging demonstrated stable disease (SD) according to RECIST 1.1 criteria, residual lesions persisted, and the biochemical trend suggested potential tumor activity. Therefore, after multidisciplinary discussion, consolidative SBRT (35 Gy in 5 fractions) was administered, primarily targeting the residual lesions in the right perihepatic space. The patient continued the original regimen until 25 months post-intervention, when blood tests revealed a significant elevation in AFP (12.20 ng/mL) and PIVKA-II (501 mAU/mL). Concurrently, CT imaging confirmed progressive disease (PD), characterized by local recurrence in the perihepatic space. The patient was subsequently diagnosed with resistance to first-line therapy and was switched to second-line therapy with regorafenib (160 mg QD) and camrelizumab (200 mg Q2W). Three months after initiating the new regimen, a favorable therapeutic response was observed, with imaging again indicating partial response (PR), accompanied by a significant decrease in tumor markers and substantial shrinkage of the lesions (**Figure 3C**). The complete diagnostic and treatment timeline is summarized in **Figure 3D**. The treatment was generally well-tolerated; except for mild diarrhea (Grade 1), no significant adverse or unanticipated events were observed. To date, this patient has achieved an OS of 30 months, PFS of 27 months, and maintained good quality of life.

**Figure 3:**
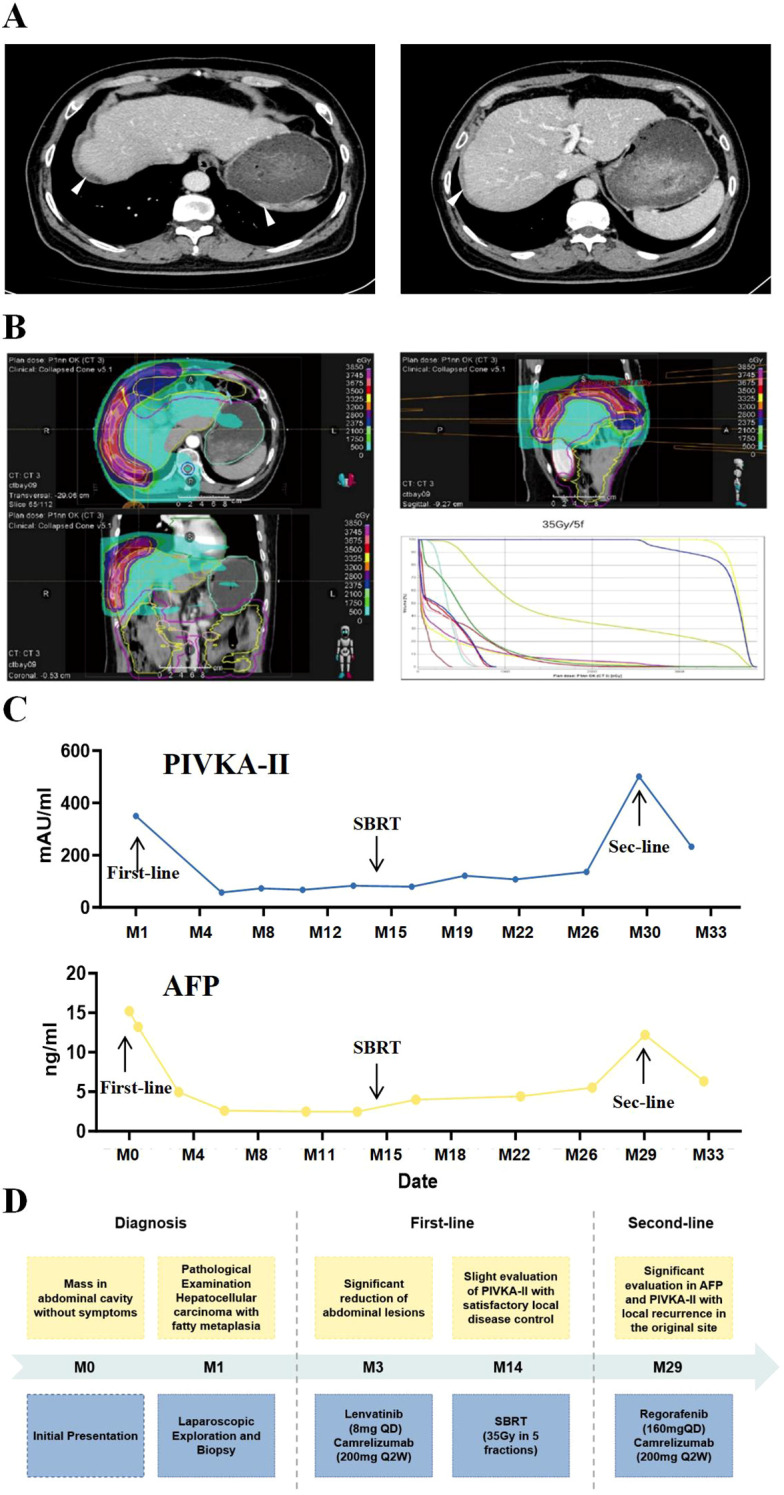
Post-treatment imaging and clinical follow-up. **(A)** Post-therapy contrast-enhanced abdominal CT demonstrates significant regression of abdominal lesions with normalization of hepatic architecture. **(B)** Supplemental radiotherapy (35 Gy in 5 fractions) targeting residual right perihepatic lesions. **(C)** Serial trends of AFP and PIVKA-II levels throughout the clinical course. **(D)** Timeline of diagnostic and therapeutic interventions. △, Tumor

## WES and RNA-seq results

WES identified recurrent somatic mutations in several cancer-associated genes, with the highest alternative allele frequencies (AltFre) observed in PAX5 (7.7%), RET (5.9%), and TP53 (5.8%), followed by the epigenetic regulators NSD3 (5.7%) and KMT2C (4.5%) (**Figure 4A**). RNA-seq analysis, using publicly available normal liver tissue data from the Gene Expression Omnibus (GEO) database (accession number: GSE241466) as controls, revealed enhanced expression of the long non-coding RNAs (lncRNAs) KCNQ1OT1 (log2FC = 13.97, FDR < 0.001) and NEAT1 (log2FC = 12.99, FDR < 0.001) in this specific tumor tissue (**Figure 4B**). These transcriptomic variations correlated with histopathological evidence of steatotic transformation within the tumor (*p* = 0.002, Fisher’s exact test). Specifically, *KCNQ1OT1* expression correlated with the intracellular lipid droplet burden (Pearson’s r = 0.62, *p* = 0.008). Hallmark pathway enrichment analysis revealed significant activation of interferon programs (IFN-γ response: NES = 2.31, p < 0.001; IFN-α response: NES = 2.04, p = 0.001), metabolic reprogramming (mTORC1 signaling: NES = 2.18, *p* < 0.001; unfolded protein response: NES = 2.08, *p* < 0.001), and oncogenic pathways (*MYC* targets: NES = 1.99, *p* < 0.001; TNF-α/NF-κB signaling: NES = 1.84, *p* = 0.002) in EHCC (**Figure 4C**). Histopathology evaluation revealed prominent immune cell infiltration, characterized by dense perivascular and interstitial lymphocyte aggregates (8–10 μm in diameter) admixed with histiocytes (15–20 μm) (**Figure 4D**), which was highly descriptive of and consistent with the transcriptomic findings identifying interferon responses as top enriched pathways (detailed sequencing protocols are available in the **Supplementary Materials**).

**Figure 4:**
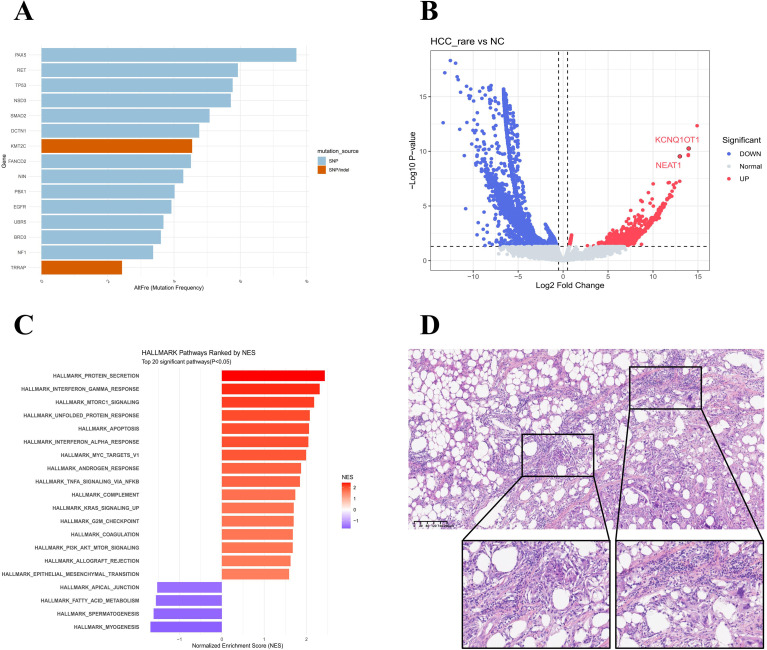
Genetic analysis of EHCC. **(A)** Mutation frequency of top altered genes identified by whole-exome sequencing (WES). Genes are ranked by mutation rate. **(B)** Volcano plot of RNA-seq data comparing EHCC samples to normal controls (NC). Significantly upregulated (red) and downregulated (blue) genes (log2FC| > 1, FDR < 0.05) are labeled. **(C)** Hallmark pathway enrichment analysis ranked by normalized enrichment score (NES). Top 10 significantly dysregulated pathways (FDR < 0.05) are shown. **(D)** HE stained tumor section demonstrating immune cell infiltration (insets: high-power views of clustered immune cells).

## Systematic review of EHCC cases

In September 2025, a systematic literature search was performed in the PubMed database (**Supplementary Figure 1A**). The search strategy employed the terms “ectopic”, “hepatocellular” and “carcinoma” restricted to title, abstract, and keywords. To ensure diagnostic rigor, only cases with a definitive histopathological diagnosis were eligible for inclusion. After excluding cases of extrahepatic metastases from occult HCC, histologically indeterminate cases, and hepatoid adenocarcinoma, 55 cases were included in the final analysis (5–10, 20–66). Although clinical details and follow-up information were not comprehensively reported in all publications, all accessible data were analyzed. Patient symptoms varied depending on the primary site of EHCC and included abdominal pain, back pain, and chest pain. The mean age was 60.85 ± 12.50 years, with a male predominance (70.91%, 39/55) over females (29.09%, 16/55). Most patients had no pre-existing liver disease. Hepatitis B virus (HBV) infection, hepatitis C virus (HCV) infection, and alcoholic chronic hepatitis were documented in only 9, 3, and 2 cases, respectively. Among these patients with chronic liver disease, cirrhosis was reported in 5 cases (**Supplementary Table 1**; **Supplementary Figure 1B**). Pretreatment AFP levels were widely evaluated; concentrations remained within the reference range in 11 cases, while mild and marked elevations were observed in 12 and 20 cases, respectively. In contrast, PIVKA-II was less frequently measured, with only 3 normal and 4 elevated results reported. EHCC was identified at multiple extrahepatic sites, in decreasing order of frequency: the abdominal cavity, pancreas, peritoneum, chest wall, retroperitoneum, extrahepatic bile ducts, adrenal gland, gallbladder, diaphragm, stomach, lung, ligament of the liver, bone, spleen, paraspinal region, and jejunum. Establishing a definitive diagnosis posed a challenge. Most patients underwent biopsy (via fine-needle aspiration or laparotomy) or surgical resection to confirm HCC. Most patients presented with a solitary lesion. Radical resection was performed in 44 cases and was associated with favorable survival outcomes. Additionally, three patients received chemotherapy and two received systemic therapy (**Supplementary Figure 1B**). The overall 1- and 3-year survival rates were 89.38% and 80.74%, respectively. Survival analysis stratified by treatment modality revealed no significant difference in OS between patients who received systemic therapy and those who did not (*p* = 0.1353, HR = 4.203, 95%CI: 0.639 - 27.670, **Supplementary Figure 1C**). Detailed information extracted from the literature is provided in **Supplementary Table 2**.

## Discussion

EHCC is an extremely rare malignancy characterized by hepatocellular carcinoma developing at extrahepatic sites without a detectable primary liver lesion. Its pathogenesis remains unclear, with proposed mechanisms including malignant transformation of ectopic liver tissue—the most accepted theory—or metastatic HCC with primary tumor regression (5, 10, 52, 67). Collan et al. (68) classified ectopic liver into four types: (1) an accessory lobe connected by a stalk; (2) a small accessory liver (10–30 g) directly attached; (3) ectopic tissue with no connection to the liver; and (4) microscopic ectopic tissue. Ectopic liver, occurring in 0.24-0.47% of individuals, is classified into four anatomical types and is most commonly found in the gallbladder, pancreas, and abdominal cavity (62, 69). Ectopic liver tissue originates from aberrant embryonic hepatoblast migration and exhibits high malignant potential (transformation rate: 12–30%) (5, 70). Its carcinogenesis, similar to conventional HCC, involves genetic mutations and chronic inflammation. Importantly, types lacking a stalk connection often lack a complete Glisson’s triad (hepatic artery, portal vein, bile duct), leading to impaired blood supply, bile drainage, cellular hypoxia, and metabolic byproduct accumulation, which collectively promote hepatocarcinogenesis (5, 50, 57). However, due to its extreme rarity and the paucity of systematic studies, the precise drivers of carcinogenesis in ectopic liver tissue remain incompletely understood. To further define the genomic profile of EHCC, we performed WES and RNA-seq, which revealed distinct molecular features characterized by somatic mutations in *PAX5*, *RET*, and *TP53*. The high mutation frequency of *PAX5*—an alteration infrequently observed in conventional HCC—suggests a unique oncogenic driver in EHCC, potentially related to its ectopic origin (71). Notably, activating RET mutations and TP53 loss-of-function variants are associated with a poor prognosis in HCC. This may explain the aggressive phenotype observed in our case, which presented with multifocal abdominal tumors at diagnosis, contrasting with the solitary lesions and prolonged post-resection survival typical of most reported cases (72). This case also exhibited a distinctive pathological feature of fatty metaplasia, characterized by lipid-laden macrophages that mimicked the histopathology of a lipomatous neoplasm. The pronounced upregulation of the lncRNAs KCNQ1OT1 and NEAT1 may participate in the localized lipid accumulation observed in this specimen, as evidenced by their strong correlation with steatotic histopathology (73, 74). However, given that transcriptomic profiles can be influenced by patient-specific background metabolic status, these molecular findings should be interpreted cautiously as observational associations within this single case, rather than a universal tumor-specific “lipid metabolic reprogramming” characteristic of EHCC itself.

Pathway enrichment analysis highlighted the concurrent activation of interferon responses (IFN-γ and IFN-α) and metabolic adaptation (via mTORC1 signaling). While this descriptive immunometabolic profiling aligns with the observed dense immune infiltrates (lymphocyte and histiocyte clusters), a definitive mechanistic link remains to be validated. In multiple studies, an interferon-rich microenvironment has been shown to favor PD-1 blockade, restore the cytotoxic capacity of CD8+ T cells, and exert significant synergistic effects in HCC (75–77). In the present case, the anti-PD-1 antibody camrelizumab was used as first-line systemic therapy and was associated with significant tumor regression and prolonged overall survival. This clinical response provides a correlationship with our hallmark pathway analysis; however, whether this represents a broadly “targetable immunogenic microenvironment” across EHCC patients requires extensive larger-cohort validation.

The diagnosis of EHCC remains challenging and it is frequently misidentified as other primary malignancies—including retroperitoneal tumors, adrenal carcinoma, or pancreatic cancer—due to its extrahepatic origin and the absence of liver imaging abnormalities. EHCC most commonly presents within the abdominal cavity, often in peritoneal or retroperitoneal locations. In contrast, pulmonary involvement—a common metastatic site for conventional HCC—is rare in primary EHCC (1). While HCC typically arises in the setting of chronic liver disease—such as HBV or HCV infection, alcoholic hepatitis, or cirrhosis—EHCC is less frequently associated with these risk factors. The prevalence of cirrhosis in EHCC is only 7.3%, and HBV infection occurs in 16.4% of cases (2, 4–6, 60, 78). Consistent with this, our patient also had no history of chronic liver disease. Biomarkers such as AFP and PIVKA-II are useful diagnostic adjuncts, but their sensitivity and specificity are limited. According to the literature, serum AFP levels exceeding 400 ng/mL are considered highly suggestive of EHCC (69). However, including the present case, 24 of 56 (42.9%) reported cases exhibited normal or only mildly elevated AFP levels. PIVKA-II levels have been less frequently analyzed; only 9 cases are documented in the literature, with 3 showing normal levels and 4 being elevated. In our case, PIVKA-II demonstrated greater sensitivity than AFP for both initial diagnosis and detection of recurrence, suggesting its diagnostic value particularly when AFP levels are unremarkable. The absence of a primary hepatic lesion on serial imaging during long-term follow-up further supported the diagnosis of EHCC. In our case, over a 30-month follow-up period, enhanced CT, gadoxetic acid–enhanced MRI (Primovist), contrast-enhanced ultrasound, and PET/CT were performed. All examinations demonstrated no abnormal lesions within the hepatic parenchyma, thereby excluding occult primary HCC with metastasis. The definitive diagnosis was established by histopathological examination. Some researchers have proposed that the diagnosis of EHCC requires the presence of non-neoplastic ectopic liver parenchyma surrounding the tumor (49). However, in the majority of reported cases, including ours, such non-neoplastic tissue is not identified, likely due to its complete replacement by tumor cells (52, 53). Furthermore, histological examination revealed a rare occurrence of hepatocellular carcinoma with fatty metaplasia, which has seldom been reported in EHCC. Although the initial diagnosis favored a lipomatous neoplasm, positive immunohistochemical staining for CK8/18, HepPar-1, GPC-3, Arg1, Ki-67, and HSP70 confirmed hepatocellular carcinoma, underscoring the critical diagnostic value of immunohistochemistry (48, 53).

Given the rarity of EHCC, evidence-based treatment guidelines are lacking. Approximately 80% of reported cases present with solitary tumors, making R0 resection feasible for localized disease. EHCC is generally confined to its site of origin, suggesting that surgical resection can be curative and is associated with prolonged overall survival (6, 41, 62, 69). Nevertheless, the prognosis for unresectable EHCC remains poor; historical case reports suggest a dismal clinical course for these patients, with an estimated median OS of approximately 8 months in the absence of standardized protocols (8–10, 41, 61, 66). Several cases received only palliative care and were lost to follow-up. For advanced orthotopic HCC, systemic agents such as sorafenib and lenvatinib have established efficacy as first-line therapies (79, 80). Furthermore, the IMbrave150 trial demonstrated the superiority of atezolizumab plus bevacizumab over sorafenib, leading to its incorporation into the Barcelona Clinic Liver Cancer (BCLC) guidelines for advanced disease (81, 82).

In our case, radical resection was not feasible. Following MDT discussion, the patient was treated with a regimen for advanced HCC, consisting of lenvatinib and camrelizumab. The tumor demonstrated objective regression (PR) after therapy initiation, and SBRT was subsequently administered to control residual abdominal lesions. Following eventual progression, second-line therapy with regorafenib further extended the patient’s survival. Notably, our survival analysis stratified by treatment modality (systemic vs. non-systemic interventions) revealed no significant difference in OS (*p* = 0.1353). These findings are likely constrained by the inherent rarity of EHCC, resulting in limited sample sizes and significant heterogeneity in historical protocols, which may obscure the true efficacy of modern, high-potency regimens. To our knowledge, this is the first reported case employing a combination of a TKI (lenvatinib) and a PD-1 inhibitor (camrelizumab) together with SBRT for unresectable EHCC. This approach was associated with prolonged PFS and OS, underscoring its potential as a promising therapeutic strategy.

This study has several limitations. First, although multi-modal imaging and biochemical profiles consistently indicated a healthy native liver, a histopathological biopsy of the hepatic parenchyma—the diagnostic gold standard—was not performed, respecting the patient’s preference for a non-invasive diagnostic course. Second, while our clinical observations are robust, we did not perform molecular characterization or functional validation to elucidate the specific biological behavior of this EHCC case; such mechanistic studies remain an essential frontier for future research. Furthermore, our systematic review was inherently constrained by the rarity of EHCC, leading to a modest sample size and significant heterogeneity in historical treatment protocols. These factors inevitably limit the generalizability of the statistical outcomes. Consequently, the accumulation of further clinical cases and the integration of genomic profiling are imperative to establish standardized, evidence-based management strategies for this rare malignancy.

## Conclusion

Confronting the challenges of rare EHCC, integrated RNA-seq and WES revealed distinct molecular features—including mutations (PAX5, RET, TP53), altered fatty acid metabolism, and interferon pathway activation. Clinically, the implementation of a novel combination of lenvatinib, camrelizumab, and SBRT was associated with a prolonged survival of 30 months, presenting a potential therapeutic option for managing this rare malignancy.

## Data Availability

The raw sequencing data (WES and RNA-seq) generated during the current study are not publicly available due to intellectual property restrictions and the preservation of patient genetic privacy, but are available from the corresponding author upon reasonable request with the permission of West China Hospital. Other clinical data supporting the findings of this study are available from West China Hospital, but restrictions apply to their availability, which were used under license for the current study. Public datasets analyzed in this study were obtained from the GEO database under accession number GSE241466.
