# Supplementary material for: Combination therapy with anti-PD-1 antibody, radiotherapy, and tyrosine kinase inhibitor for unresectable primary ectopic hepatocellular carcinoma: a case report with genomic profiling and literature review
Source: Front Immunol. 2026 Jul 14;17:1746990. doi: 10.3389/fimmu.2026.1746990 (PMC13408394; doi:10.3389/fimmu.2026.1746990)
Supplement: Supplementary file 1 [file DataSheet1.docx]

## Supplementary Materials

### Supplementary Methods

**Immunohistochemical staining**

Immunohistochemical staining was performed on 4-μm-thick human FFPE sections. Following deparaffinization, heat-induced antigen retrieval, and serum blocking, the slides were incubated overnight at 4°C with primary antibodies purchased from OriGene Technologies (Rockville, MD, USA): anti-HepPar-1 (clone OCH1E5, 1:100), anti-GPC-3 (polyclonal, 1:100), anti-CK8&18 (clone 5D3, 1:20), anti-HSP70 (clone OTI3C6, 1:150), anti-Arg-1 (clone OTIRA0042, 1:100), and anti-Ki-67 (clone UMAB107, 1:200). Sections were then incubated with an HRP-polymer secondary antibody (OriGene) and visualized with DAB. Finally, whole-slide images were acquired using the SLIDEVIEW VS200 slide scanner (Olympus, Tokyo, Japan).

**Whole-Exome Sequencing (WES) and Bioinformatics Pipeline**

Genomic DNA was extracted from the patient’s laparoscopic biopsy formal-fixed paraffin-embedded (FFPE) blocks. Under strict histopathological guidance, matched adjacent non-tumor stromal elements identified at the margins of the biopsy tissue within the same block were isolated to serve as the normal germline baseline. A total of 200 ng of qualified DNA was fragmented and prepared using the VAHTS Universal Plus DNA Library Prep Kit for Illumina V2 (Vazyme). Target region enrichment was performed using the Agilent SureSelect Human All Exon V6 kit, followed by paired-end 150 bp sequencing on the Illumina HiSeq X Ten platform (Annoroad Gene Tech). Clean reads were aligned to the human reference genome (hg19) using BWA, with duplicates marked by Picard and local realignment performed via GATK. Somatic SNVs and InDels were detected using MuTect2, copy number variations (CNVs) were characterized via Control-FREEC, and structural variations were detected with Delly. Somatic variants were annotated using ANNOVAR against public databases.

**RNA-Seq Processing and Differential Expression Analysis**

Total RNA was extracted from the fresh frozen tumor biopsy fragment. Due to the critical scarcity of non-tumor components in the small biopsy sample, all adjacent tissues were entirely consumed for routine diagnostic pathology (FFPE embedding) and were unfeasible for high-quality RNA-seq. Consequently, a public dataset of normal liver tissues (GSE241466) was retrieved from the GEO database as an alternative transcriptomic baseline. The RNA-seq libraries were sequenced on the Illumina platform. Comparative transcriptomic analysis was conducted using the limma package (version 3.58.1) in R (version 4.4.3). Lowly expressed genes (CPM < 1 in more than 1 sample) were filtered out. Read counts were normalized using the voom method, and differential expression was assessed via empirical Bayes moderation. Multiple testing corrections were applied using the Benjamini-Hochberg FDR procedure. Significant differentially expressed genes (DEGs) were defined based on an adjusted p-value (padj) < 0.05 and an absolute fold-change > 1.5 (|log₂FC| > 0.585). Volcano plots were visualized using the ggplot2 package (version 3.5.1). Additionally, Gene Set Enrichment Analysis (GSEA) using MSigDB Hallmark gene sets was performed via the fgsea package, with significant pathways (p < 0.05) ranked by Normalized Enrichment Score (NES).

### Supplementary Figure

**Supplementary Figure 1** Summary of literature review. **(A)** PRISMA flow diagram of the literature review. **(B)** Summary of reported EHCC features in the literatures including: predominant site of EHCC, number of tumor, pre-existing liver diseases, pretreatment AFP levels, and therapeutic interventions. **(C)** The Kaplan-Meier curves of OS for the systemic treatment group and the non-systemic treatment group.

###
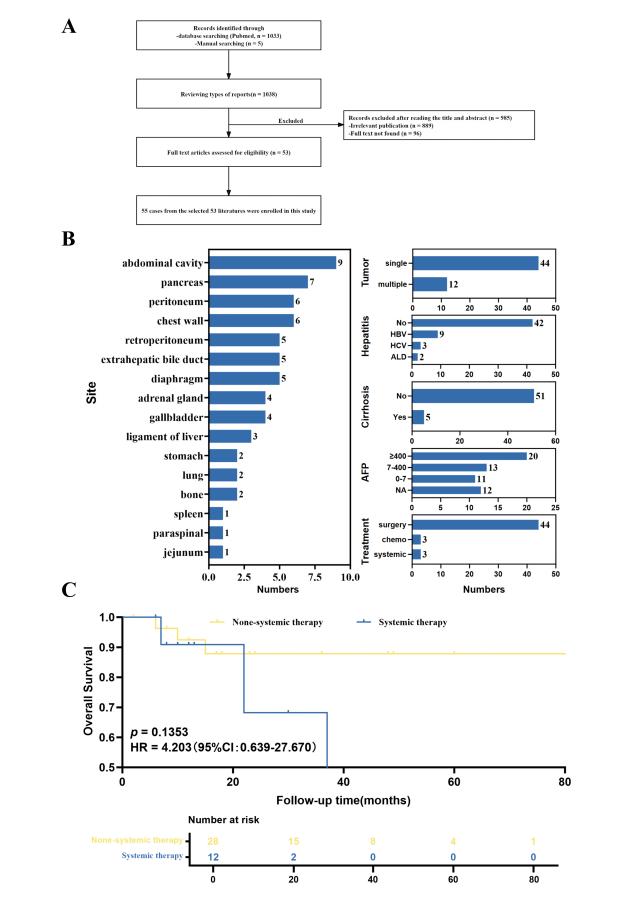


### Supplementary Tables

**Supplementary Table 1** Summary of the literature review.

| Variable |  | Value |
| --- | --- | --- |
| Age |  | 60.85±12.50 |
| Gender |  |  |
|  | Female | 16(29.09%) |
|  | Male | 39(70.91%) |
| Hepatitis |  |  |
|  | No | 41(74.55%) |
|  | ALD | 2(3.64%) |
|  | HBV | 9(16.36%) |
|  | HCV | 3(5.45%) |
| Cirrhosis |  |  |
|  | No | 50(90.91%) |
|  | Yes | 5(9.09%) |
| Tumor number |  |  |
|  | single | 44(80.00%) |
|  | multiple | 11(20.00%) |
| AFP |  |  |
|  | 0-7 | 11(20.00%) |
|  | 7-400 | 12(21.82%) |
|  | ≥400 | 20(36.36%) |
|  | NA | 12(21.82%) |
| PIVKA-II |  |  |
|  | normal | 3(5.45%) |
|  | elevated | 4(7.27%) |
|  | NA | 48(87.27%) |
| Largest tumor diameter |  | 7.5(4.15-11.9) |
| Follow-up months |  | 17(12.00-36.75) |
| Progressive patients |  | 13(31.71%) |
| 1-year survival rate |  | 89.38% |
| 3-year survival rate |  | 80.74% |

HBV, Hepatitis B virus; HCV, Hepatitis C virus; ALD, Alcoholic Liver Disease; AFP, Alpha-fetoprotein; PIVKA-II, Protein Induced by Vitamin K Absence or Antagonist II.

**Supplementary Table S2** summary of 56 EHCC cases identified by literatures and the present case.

| **Center** | **Report year, Country** | **age, gender** | **Number** | **Site** | **Liver disease** | **Tumor size (cm)** | **AFP (ng/ml)** | **PIVKA-ⅠⅠ (mAU/ml)** | **Diagnosis achieved by** | **Treatment** | **Follow-up duration (months)** | **Status at follow-up** |
| --- | --- | --- | --- | --- | --- | --- | --- | --- | --- | --- | --- | --- |
| Kanazawa University Cancer Research Institute Hospital | 1988, Japan | 64, male | multiple | peritoneum,diaphragm,Douglas fossa and major omentum | No | NA | 117000 | NA | Resection | Resection + Chemo (adriamycin,mitomycin and neocarcinostatin) | 37 | DEAD |
| National Cancer Center Hospital | 1994, Japan | 57, male | single | left diaphragm | No | 5×4 | 2207 | NA | Resection | Resection | 96 | NED |
| Yong Dong Severance Hospital | 1996, Korea | 36, male | single | upper common hepatic duct | HBV,cirrhosis | 3×2 | 22.1 | NA | Resection | Resection | 12 | NED |
| Ohmuta Municipal Hospital | 1999, Japan | 64, male | single | stomach | No | 4.5×2×2 | 4900 | NA | Resection | Resection | 15 | DEAD |
| Saint André Hospital | 1999, France | 65, female | single | left ligament between the left lobe and the diaphragm | No | 6×5.5 | NA | NA | Resection | Resection | NA | LTFU |
| Hospital Beaujon | 2001, France | 66, male | single | left chest wall | HCV | 17×10×8 | negative | NA | Resection | Resection | 36 | NED |
| Guro Hospital | 2003, Korea | 43, female | single | between the spleen and left diaphragm | HBV | 10×7 | NA | NA | Resection | Resection → RFA (7 mo post-op) | 23 | AWD |
| Molinette Hospital | 2004, Italy | 54, female | single | gallbladder | No | 9×8×7 | NA | NA | Resection | Resection | 48 | NED |
|  |  | 34, female | single | subdiaphragmatic region,tightly adherent to the spleen | No | 10×6 | NA | NA | Resection | Resection + Chemo | 48 | AWD |
|  |  | 62, male | single | between the diaphragm and the spleen | No | 9×7 | 4000 | NA | Resection | Resection | 48 | NED |
| Yamaguchi University School of Medicine | 2005, Japan | 72, female | single | extrahepatic bile duct | No | 2.7×2.1 | NA | NA | Resection | Resection | 12 | NED |
| "G. Papanikolaou" General Hospital | 2005, Greece | 76, male | single | left adrenal gland | HBV,cirrhosis | 10 | 75.6 | NA | Resection | Resection → TAE (7 mo post-op) | 10 | DEAD |
| Mayo Clinic College of Medicine | 2006, USA | 35, male | single | upper abdomen | No | NA | negative | NA | Biopsy | Chemo (etoposide + epirubicin; qMonthly) | 7 | DEAD |
| Hanyang University College of Medicine | 2006, Korea | 51, male | single | left upper chest wall | HBV,cirrhosis | 14×10×7.5 | 308.3 | NA | Resection | Resection → RT + Chemo (adriamycin, 4 mo post-op) | 12 | AWD |
| Otsu Red Cross Hospital | 2006, Japan | 72, male | single | jejunum | No | 14×11×10 | 99100 | negative | Resection | Resection → TACE (2 mo post-op) | 12 | AWD |
| University of Florida,College of Medicine | 2007, USA | 58, male | single | the distal body of the pancreas | No | 3.3×2.5×2.5 | NA | NA | Resection | Resection | 15 | NED |
| National Taiwan University Hospital | 2007, China | 81, female | single | peritoneum in lower abdomen | No | 15×10 | ＞87500 | NA | Biopsy | Resection | NA | LTFU |
| Tri-Service General Hospital | 2007, China | 62, female | single | diaphragm | No | 16×14×5 | 45000 | NA | Resection | Resection → Adjuvant chemo ×2 cycles (cisplatin + etoposide (VP-16) + bleomycin) | 8 | NED |
| Dokkyo University Hospital | 2007, Japan | 56, male | single | the tail of the pancreas | No | 6.3×6.2 | NA | NA | Resection | Resection | 36 | NED |
| University of Pittsburgh Medical Center | 2007, USA | 62, male | single | left chest wall | No | 15 | 16425 | NA | Biopsy | Neoadjuvant chemo + RT → Resection | NA | LTFU |
| Guro Hospital | 2008, Korea | 59, male | single | between the diaphragm and the spleen | No | 4.6×3.6×3.5 | negative | NA | Resection | Resection | NA | LTFU |
| Heinrich-Heine-University Dusseldorf | 2009, Germany | 75, male | single | the extrahepatic bile duct | No | NA | 7.8 | NA | Biopsy | Resection | NA | LTFU |
| Radboud University Nijmegen Medical Centre | 2010, Netherland | 46, male | multiple | the upper abdomen with multiple enlarged lymph nodes in the mesentery | No | NA | 24000(kU/L) | NA | Biopsy | Sorafenib (400 mg BID) | 6 | NED |
| Chhatrapati Shahuji Maharaj Medical University | 2010, India | 60, male | single | left suprarenal region | HBV | 8×8×8 | 35 | NA | Resection | Resection | 6 | DEAD |
| Osaka Medical Center for Cancer and Cardiovascular Diseases | 2010, Japan | 59, female | single | the left triangular ligament of the liver | No | 2 | 2508 | normal | Resection | Resection | 18 | NED |
| Evaggelismos General Hospital | 2011, Greece | 68, male | multiple | the right serratus anterior muscle,with strong adhesions to the parietal pleura;the left parietal bone | No | 7 | positive | NA | Resection | Resection | 24 | NED |
| Oita University Faculty of Medicine | 2011, Japan | 64, male | multiple | the left diaphragm and lung | No | NA | 84865 | 1384 | Biopsy | NA | NA | LTFU |
| Fukuoka University School of Medicine | 2012, Japan | 42, male | multiple | the peritoneum and mesentery | No | 1 | 241 | 69349 | Biopsy | Chemo | NA | LTFU |
| John Wayne Cancer Institute | 2013, USA | 61, female | single | the tail of the pancreas | No | 5 | 4.9 | NA | Biopsy | Resection | 60 | NED |
| University Hospital Ostrava | 2013, Czech Republic | 59, male | single | the upper pole of the spleen | Alcoholic liver disease | 10×8×6 | negative | NA | Biopsy | Resection → Adjuvant targeted therapy (sorafenib) | NA | LTFU |
| Hospital San Juan de Dios del Aljarafe | 2014, Spain | 49, female | single | gallbladder | No | 12 | 13785 | NA | Resection | Resection | 36 | NED |
| Recep Tayyip Erdogan University Training and Research Hospital | 2014, Turkey | 72, male | multiple | the retroperitoneal | No | 6.4×6.0, 22 | ＞20000 | NA | Biopsy | Refused treatment | NA | LTFU |
| University of Oslo | 2015, Norway | 64, female | single | the upper left abdominal quadrant | No | 3.5×2.5×1 | 200 | NA | Resection | Resection → Re-resection (post-recurrence) | 48 | AWD |
| Inje University Busan Paik Hospital | 2015, Korea | 65, male | single | left subphrenic region | No | 3.8×3.2×1.2 | NA | NA | Resection | Resection | 17 | NED |
| Xinqiao Hospital | 2016, China | 63, male | multiple | lung,mediastinal,the fundus of stomach,cardia,portal fissure and abdominal aortas | HBV,cirrhosis | 1.7×1.7,6.4×3.2,4.6×2.2 | 24793 | NA | Biopsy | Right lung wedge resection → Sorafenib 400 mg BID + Jinlong (TCM) + ubenimex | 13 | NED |
| Medical University of Lodz | 2017, Poland | 77, male | single | the tail of the pancreas | No | 2.5 | negative | NA | Resection | Resection | 24 | NED |
| West China Hospital | 2017, China | 44, female | multiple | adjacent to the pancreas | No | 5×4,4×3 | 1200 | NA | Resection | Resection | 17 | NED |
| Baylor College of Medicine | 2017, USA | 82, male | single | the left anterior lower hemithorax | No | 13.3×10 | NA | NA | Biopsy | Palliative therapy | NA | LTFU |
| University of Arkansas for Medical Sciences | 2017, USA | 69, male | single | choledochal cyst | No | NA | 2.9 | NA | Biopsy | Palliative therapy | NA | LTFU |
| Sun Yat-sen University,the First Affiliated Hospital | 2017, China | 54, male | single | the gallbladder | HBV,cirrhosis | NA | 3724.75 | NA | Resection | Resection | NA | LTFU |
| Sir Run Run Shaw Hospital | 2017, China | 56, male | multiple | upper abdomen,adhered to the spleen,omentum,and left diaphragm | No | 30×20×15,20×15×10 | 8.03 | NA | Resection | Resection → Adjuvant therapy → Palliative surgery | 22 | DEAD |
| Navarra Hospital Complex | 2019, Spain | 68, female | multiple | the juxtacolic greater omentum next to the transverse colon | No | 8×6×4 | 651 | NA | Resection | Resection | 24 | NED |
| Kumamoto University | 2020, Japan | 81, female | single | the retroperitoneal | HCV | 7.5×6.5×3.5 | 30.1 | 17 | Biopsy | Resection | 8 | NED |
| Saiseikai Kanazawa Hospital | 2022, Japan | 82, male | single | thoracic vertebrae | No | NA | 3.1 | 430 | Biopsy | TAE + RT | 8 | DEAD, pneumonia |
| Montpellier Cancer Institute | 2022, France | 59, female | multiple | peritoneum | No | NA | NA | NA | Biopsy | Chemo → CRS/HIPEC | NA, liver metastasis | LTFU |
| New York Medical College | 2022, USA | 71, male | single | the right adrenal region and the inferior vena cava | No | 9.1×8.2×8.6 | 1.87 | NA | Resection | Resection + Lenvatinib | 10 | AWD |
| Zhejiang University School of Medicine,Affiliated Hangzhou First People's Hospital | 2022, China | 59, male | single | the tail of the pancreas | No | 1.8×1.4 | 3.1 | NA | Resection | Resection | 84 | NED |
| Portuguese Institute of Oncology of Porto Francisco Gentil | 2023, Portugal | 55, male | single | the body of the pancreas | No | 2.9×2.5×2.8 | NA | NA | Resection | Resection | 49 | NED |
| Handan Central Hospital | 2023, China | 47, male | single | the left subdiaphragm,between the spleen and stomach | No | 6.3×9.3 | 194.38 | NA | Resection | Resection → HAIC(×2,1-mo interval) | 2 | NED |
| Hunan Provincial People’s Hospital | 2023, China | 61, male | single | right adrenal region | HBV | 11.8×11×8.3 | 23.69 | NA | Resection | Resection → Sintilimab + Lenvatinib | 12 | NED |
| Yeungnam University,College of medicine | 2023, Korea | 61, male | single | left subphrenic space adjacent to the stomach | No | 7.3×6.8×4.5 | negative | 83.99 | Resection | Resection | 12 | NED |
| Tianjin Medical University Cancer Institute & Hospital | 2023, China | 30, male | single | right adrenal region | HBV | 12×10×8 | 51583 | NA | Resection | Resection → Regorafenib (6 mo post-op) | 12 | NED |
| Sindh Institute of Urology and Transplantation | 2023, Pakistan | 62, male | single | the left lumbosacral paravertebral region | HCV | NA | 1560000 | NA | Biopsy | RT + Sorafenib | NA | LTFU |
| Meiwa Hospital | 2023, Japan | 74 male | single | the gallbladder | Alcoholic chronic hepatitis | 1 | NA | NA | Resection | Resection | 60 | NED |
| Saint Joseph University,Hotel Dieu de France Hospital | 2024, Lebanon | 79 female | single | the pericaval retroperitoneal | No | 5.9 | 4655 | NA | Biopsy | NA | NA | LTFU |
| West China Hospital | 2025, China | 64, male | multiple | the diaphragm,hepatic round ligament,anterior abdominal wall,lateral abdominal wall and pelvic cavity | No | 12.2×1.5  (the lagest) | 13.2 | 350 | Biopsy | Lenvatinib + Camrelizumab + SBRT | 30 | AWD |

HBV, Hepatitis B virus; HCV, Hepatitis C virus; AFP, Alpha-fetoprotein; PIVKA-II, Protein Induced by Vitamin K Absence or Antagonist II; Chemo, chemotherapy; RFA, Radiofrequency Ablation; SBRT, Stereotactic Body Radiation Therapy; RT, Radiation Therapy; HAIC, Hepatic Arterial Infusion Chemotherapy; CRS/HIPEC, Cytoreductive Surgery / Hyperthermic Intraperitoneal Chemotherapy; TAE, Transarterial Embolization; TACE, Transarterial Chemoembolization; AWD, Alive With Disease; NED, No Evidence of Disease; LTFU, Lost to Follow-up.
